# 2-Butyl-11-phenyl-5,10-dihydro-1*H*-benzo[*e*]imidazo[1,5-*a*][1,4]diazepine-1,3(2*H*)-dione

**DOI:** 10.1107/S1600536810005477

**Published:** 2010-02-13

**Authors:** Gary S. Nichol, Steven Gunawan, Justin Dietrich, Christopher Hulme

**Affiliations:** a Department of Chemistry and Biochemistry, The University of Arizona, 1306 E. University Boulevard, Tucson, AZ 85721, USA; b Southwest Center for Drug Discovery and Development, College of Pharmacy, BIO5 Institute, University of Arizona, Tucson, AZ 85721, USA

## Abstract

The title compound, C_21_H_21_N_3_O_2_, was obtained following a five-step synthetic procedure yielding weakly diffracting rod and needle-shaped crystals which crystallized concomitantly. Structural analysis of a rod-shaped crystal showed that the central seven-membered heterocyclic ring adopts a conformation that is perhaps best described as a distorted boat, with the H-bearing (CH_2_ and NH) atoms lying well out of the least-squares mean plane fitted through the other five atoms in the ring (r.m.s. deviation 0.075 Å). In the crystal, the compound packs as a twisted chain, which propagates along the *b* axis by means of an *R* ^1^_2_(6) motif formed by one of the carbonyl O atoms acting as a bifurcated acceptor in an N—H⋯O and C—H⋯O inter­action. No diffraction was observed from the needle-shaped crystals.

## Related literature

For background to the synthetic procedure, see: Hulme & Gore (2003 ▶); Hulme *et al.* (2000 ▶). For graph-set analysis of hydrogen-bond networks, see: Bernstein *et al.* (1995 ▶).
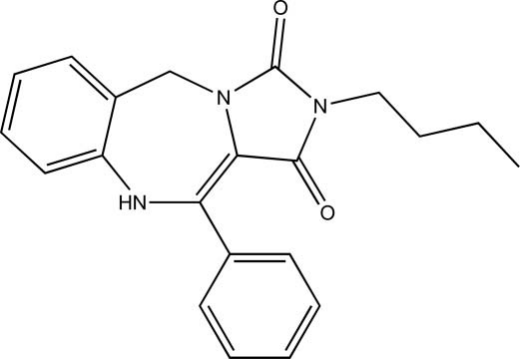


## Experimental

### 

#### Crystal data

C_21_H_21_N_3_O_2_
*M* *_r_* = 347.41
Monoclinic, 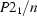

*a* = 12.192 (4) Å
*b* = 7.638 (2) Å
*c* = 18.514 (6) Å
β = 95.494 (5)°
*V* = 1716.1 (9) Å^3^
*Z* = 4
Mo *K*α radiation
μ = 0.09 mm^−1^
*T* = 100 K
0.29 × 0.14 × 0.08 mm

#### Data collection

Bruker Kappa APEXII DUO CCD diffractometer
Absorption correction: numerical (*SADABS*; Sheldrick, 1996 ▶) *T*_min_ = 0.975, *T*_max_ = 0.993
14047 measured reflections
2724 independent reflections
1908 reflections with *I* > 2σ(*I*)
*R*_int_ = 0.067
θ_max_ = 24.1°

#### Refinement

*R*[*F* ^2^ > 2σ(*F* ^2^)] = 0.065
*wR*(*F* ^2^) = 0.181
*S* = 1.03
2724 reflections
239 parameters
H atoms treated by a mixture of independent and constrained refinement
Δρ_max_ = 0.71 e Å^−3^
Δρ_min_ = −0.29 e Å^−3^

### 

Data collection: *APEX2* (Bruker, 2007 ▶); cell refinement: *SAINT* (Bruker, 2007 ▶); data reduction: *SAINT*; program(s) used to solve structure: *SHELXTL* (Sheldrick, 2008 ▶); program(s) used to refine structure: *SHELXTL*; molecular graphics: *ORTEP-3 for Windows* (Farrugia, 1997 ▶) and *Mercury* (Macrae *et al.*, 2008 ▶); software used to prepare material for publication: *SHELXTL* and local programs.

## Supplementary Material

Crystal structure: contains datablocks I, global. DOI: 10.1107/S1600536810005477/bh2272sup1.cif

Structure factors: contains datablocks I. DOI: 10.1107/S1600536810005477/bh2272Isup2.hkl

Additional supplementary materials:  crystallographic information; 3D view; checkCIF report

## Figures and Tables

**Table 1:** Hydrogen-bond geometry (Å, °)

*D*—H⋯*A*	*D*—H	H⋯*A*	*D*⋯*A*	*D*—H⋯*A*
N3—H3*N*⋯O1^i^	0.86 (4)	2.10 (4)	2.944 (3)	165 (3)
C8—H8⋯O1^i^	0.95	2.57	3.326 (4)	136

Symmetry code: (i) 
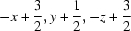
.

**Fig. 1.**
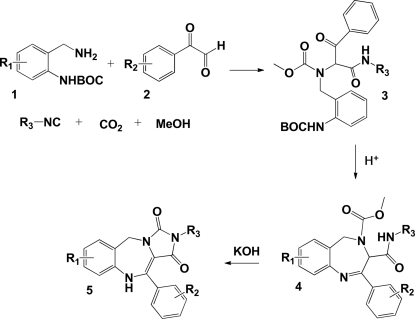
Synthetic route to 5.

**Fig. 2.**
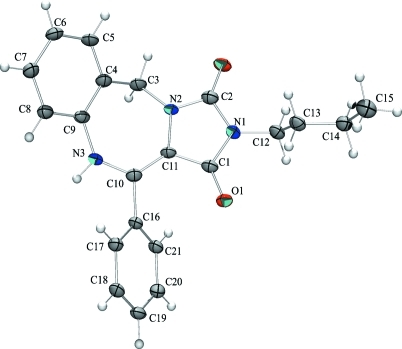
The molecular structure of 5 with displacement ellipsoids at the 50% probability level.

**Fig. 3.**
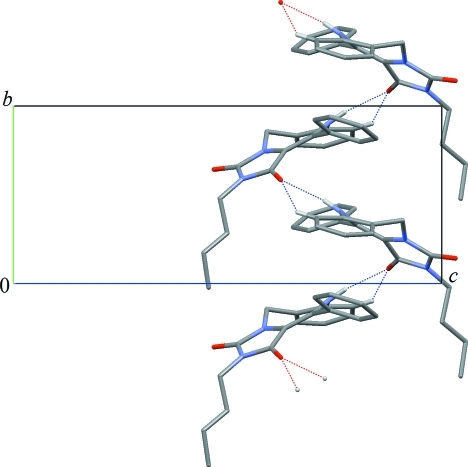
An a-axis projection of 5 showing the twisted hydrogen-bonded chain (blue dotted lines; red dotted lines indicate continuation).

## supplementary crystallographic information

### Comment

We recently investigated a three step solution phase protocol for the synthesis of arrays of tricyclic fused hydantoin-benzodiazepines as part of broader research on multi-component reactions (Figure 1). Interestingly, the major product of this unique synthetic route was the tautomer 5 derived from the originally desired product 4, the structure being confirmed by X-ray crystallography. The methodology employs *ortho*-*N*-Boc benzylamines 1 and phenylglyoxaldehydes 2 in the rarely used five component Ugi reaction with CO_2_ to assemble desired diversity in product 3 (Hulme *et al.*, 2000). Acid treatment unmasks an internal amino nucleophile and promotes rapid formation of the diazepine ring of generic structure 4. Subsequent base treatment employs the amidic NH of the Ugi scaffold as a second internal nucleophile promoting hydantoin formation and an unexpected 1,3-H shift to give 5. As such the methodology represents an example of a post-condensation Ugi modification (Hulme & Gore, 2003) that employs two internal nucleophiles in distinct operations, generating a novel scaffold of high complexity in three succinct functional operations.

Two types of crystals were formed: very fine yellow needles together with a few slightly larger rod-shaped pale yellow crystals. The needles did not give any measurable diffraction and the rod crystals showed weak diffraction with 60 second exposure times; a resolution cutoff of 0.87Å was applied to the dataset. The identity of the needle crystals was not established. The molecular structure of 5 is shown in Figure 2. Molecular dimensions are unexceptional. The amine hydrogen atom was located in a difference Fourier map and its presence is confirmed by participation in hydrogen bonding discussed below. The central 7-membered heterocyclic ring adopts a conformation that is perhaps best described as a distorted boat with the H-bearing (C3 and N3) atoms lying well out of a least squares mean plane fitted through the other five atoms in the ring [r.m.s. deviation 0.075 Å; C3 deviates by 0.679 (5) Å and N3 deviates by 0.301 (4) Å]. The compound packs as a twisted chain which propagates along the *b* axis by means of an *R^1^_2_*(6) motif (Bernstein *et al.*, 1995) formed by one of the carbonyl oxygen atoms acting as bifurcated acceptor in an N–H···O and C–H···O interaction (Figure 3).

### Experimental

*Ugi reaction (For R_1_, R_2_=H, R_3_= n-butyl)*

CO_2_ gas was bubbled through a stirring solution of MeOH for 25 minutes to generate methyl carbonic acid. In a separate 25 ml flask, phenyl glyoxal 2 (226 mg, 1.687 mmol) was added to BOC-2-aminobenyzlamine 1 (250 mg, 1.125 mmol). Methyl carbonic acid (10 ml) and *N*-butyl isonitrile (0.237 ml, 2.252 mmol) were then added to the latter flask. The reaction was stirred at room temperature under an atmosphere of CO_2_ for 16 h. The solvent was evaporated *in vacuo* and the crude product purified with a Biotage Isolera4^TM ^system (hexane/EtOAc 10-30%) to afford the Ugi product 3 (218 mg, 0.438 mmol, 39%) as a yellow oil.

*De-BOC and Cyclization*

3 (0.180 g, 0.362 mmol) was treated with a 5 ml 10% TFA solution in 1,2-dichloroethane which was irradiated in a Biotage InitiatorTM at 80°C for 20 min. The resulting orange solution was washed with 1M NaHCO_3_ (4 × 2.5 ml) and the organic layer dried (Na_2_SO_4_), filtered and evaporated *in vacuo*. MeOH (1.5 ml), THF (0.75 ml), H_2_O (0.5 ml) were added to the crude product 4 (0.102 g, 0.269 mmol) followed by a 1 g/1 ml solution of KOH in H_2_O (0.03 ml). The solution was irradiated at 100°C for 20 min and resultant orange solution partitioned between EtOAc (5 ml) and 1M NaHCO_3_ (5 ml). The organic layer was dried (Na_2_SO_4_), filtered and evaporated *in vacuo*. Final crude product was purified with a Biotage Isolera4^TM^ (hexane/EtOAc 30%) to afford the final product 5 (0.074 g, 0.214 mmol, 80%) as a yellow solid. FT-ICR calculated for C_21_H_22_N_3_O_2_ [M+H]^+^: 348.1707, found: 348.1707.

### Refinement

A resolution cutoff of 0.87 Å was applied to the dataset due to unobserved diffraction beyond this point. Nevertheless the N—H hydrogen atom was located in a difference Fourier map and the N—H distance freely refined to 0.86 (4) Å with *U*_iso_(H) = 1.2 *U*_eq_(N). C—H atoms were refined with *U*_iso_(H) = 1.5 *U*_eq_(C) (methyl) or *U*_iso_(H) = 1.2 *U*_eq_(C) (all others) with constrained C—H distances in the range 0.95–0.99 Å. The largest residual peak, 0.7 e.Å^-3^, is approximately 1.53 Å from C21.

### Crystal data

**Table:**

C_21_H_21_N_3_O_2_	*F*(000) = 736
*M**_r_* = 347.41	*D*_x_ = 1.345 Mg m^−^^3^
Monoclinic, *P*2_1_/*n*	Mo *K*α radiation, λ = 0.71073 Å
Hall symbol: -P 2yn	Cell parameters from 2525 reflections
*a* = 12.192 (4) Å	θ = 2.2–24.8°
*b* = 7.638 (2) Å	µ = 0.09 mm^−^^1^
*c* = 18.514 (6) Å	*T* = 100 K
β = 95.494 (5)°	Rod, yellow
*V* = 1716.1 (9) Å^3^	0.29 × 0.14 × 0.08 mm
*Z* = 4	

### Data collection

**Table:**

Bruker Kappa APEXII DUO CCD diffractometer	2724 independent reflections
Radiation source: fine-focus sealed tube with Miracol optics	1908 reflections with *I* > 2σ(*I*)
graphite	*R*_int_ = 0.067
φ and ω scans	θ_max_ = 24.1°, θ_min_ = 1.9°
Absorption correction: numerical (*SADABS*; Sheldrick, 1996)	*h* = −14→14
*T*_min_ = 0.975, *T*_max_ = 0.993	*k* = −8→5
14047 measured reflections	*l* = −21→21

### Refinement

**Table:**

Refinement on *F*^2^	Primary atom site location: structure-invariant direct methods
Least-squares matrix: full	Secondary atom site location: difference Fourier map
*R*[*F*^2^ > 2σ(*F*^2^)] = 0.065	Hydrogen site location: difference Fourier map
*wR*(*F*^2^) = 0.181	H atoms treated by a mixture of independent and constrained refinement
*S* = 1.03	*w* = 1/[σ^2^(*F*_o_^2^) + (0.1146*P*)^2^ + 0.6555*P*] where *P* = (*F*_o_^2^ + 2*F*_c_^2^)/3
2724 reflections	(Δ/σ)_max_ = 0.001
239 parameters	Δρ_max_ = 0.71 e Å^−^^3^
0 restraints	Δρ_min_ = −0.29 e Å^−^^3^
0 constraints	

### Special details

**Table:**

Experimental. ^1^H NMR (300 MHz, CDCl_3_) δ ppm 0.89 (t, *J* = 7.2 Hz, 3H), 1.28 (m, 2H), 1.56 (m, 2H), 3.49 (t, *J* = 7.3 Hz, 2H), 4.98 (s, 2H), 5.95 (s, 1H), 6.89 (d, *J* = 7.8 Hz, 1H), 7.06 (t, *J* = 7.2 Hz, 1H), 7.28 (t, *J* = 7.7 Hz, 1H), 7.34 (d, *J* = 7.5 Hz, 1H), 7.52 (m, 5H).13C NMR (75 MHz, CDCl_3_) δ ppm 14.1, 20.5, 30.7, 39.0, 45.7, 109.4, 120.7, 123.9, 126.4, 129.1, 129.6, 129.7, 130.5, 130.6, 134.6, 135.4, 142.5, 153.7, 161.9.

### Fractional atomic coordinates and isotropic or equivalent isotropic displacement parameters (Å^2^)

**Table:**

	*x*	*y*	*z*	*U*_iso_*/*U*_eq_	
O1	0.88328 (17)	0.0841 (3)	0.87585 (11)	0.0267 (6)	
O2	0.61570 (19)	0.1425 (3)	1.03172 (11)	0.0346 (6)	
N1	0.7648 (2)	0.0891 (4)	0.96528 (13)	0.0249 (6)	
N2	0.62702 (19)	0.2468 (3)	0.91468 (13)	0.0221 (6)	
N3	0.6120 (2)	0.4044 (4)	0.76450 (14)	0.0253 (7)	
H3N	0.625 (3)	0.466 (5)	0.7271 (19)	0.030*	
C1	0.7968 (3)	0.1375 (4)	0.89869 (16)	0.0243 (8)	
C2	0.6618 (3)	0.1580 (5)	0.97655 (17)	0.0282 (8)	
C3	0.5292 (3)	0.3561 (5)	0.90814 (17)	0.0290 (8)	
H3A	0.5519	0.4803	0.9130	0.035*	
H3B	0.4843	0.3285	0.9485	0.035*	
C4	0.4598 (2)	0.3329 (5)	0.83796 (17)	0.0272 (8)	
C5	0.3498 (3)	0.2811 (5)	0.83711 (18)	0.0308 (8)	
H5	0.3222	0.2501	0.8816	0.037*	
C6	0.2800 (3)	0.2733 (5)	0.77408 (18)	0.0316 (8)	
H6	0.2060	0.2351	0.7752	0.038*	
C7	0.3188 (3)	0.3216 (5)	0.70925 (18)	0.0295 (8)	
H7	0.2707	0.3223	0.6658	0.035*	
C8	0.4285 (3)	0.3690 (4)	0.70802 (17)	0.0274 (8)	
H8	0.4551	0.4017	0.6634	0.033*	
C9	0.4995 (2)	0.3694 (4)	0.77089 (17)	0.0241 (8)	
C10	0.7038 (2)	0.3312 (4)	0.80057 (16)	0.0239 (8)	
C11	0.7095 (2)	0.2491 (4)	0.86567 (16)	0.0215 (7)	
C12	0.8299 (3)	−0.0207 (5)	1.01778 (16)	0.0291 (8)	
H12A	0.8060	0.0008	1.0666	0.035*	
H12B	0.9083	0.0136	1.0189	0.035*	
C13	0.8194 (3)	−0.2160 (5)	1.00075 (16)	0.0290 (8)	
H13A	0.7411	−0.2509	0.9997	0.035*	
H13B	0.8435	−0.2381	0.9520	0.035*	
C14	0.8874 (3)	−0.3268 (5)	1.05583 (17)	0.0297 (8)	
H14A	0.8702	−0.2937	1.1052	0.036*	
H14B	0.9666	−0.3032	1.0523	0.036*	
C15	0.8653 (3)	−0.5210 (5)	1.04422 (19)	0.0377 (9)	
H15A	0.7890	−0.5471	1.0531	0.057*	
H15B	0.9157	−0.5887	1.0779	0.057*	
H15C	0.8768	−0.5525	0.9942	0.057*	
C16	0.8047 (2)	0.3506 (4)	0.76174 (16)	0.0231 (7)	
C17	0.8030 (2)	0.2989 (5)	0.68966 (16)	0.0256 (8)	
H17	0.7377	0.2506	0.6654	0.031*	
C18	0.8963 (3)	0.3177 (5)	0.65308 (16)	0.0279 (8)	
H18	0.8943	0.2830	0.6037	0.033*	
C19	0.9920 (3)	0.3863 (5)	0.68772 (17)	0.0292 (8)	
H19	1.0559	0.3979	0.6625	0.035*	
C20	0.9944 (3)	0.4382 (4)	0.75934 (17)	0.0272 (8)	
H20	1.0601	0.4852	0.7835	0.033*	
C21	0.9005 (2)	0.4217 (4)	0.79611 (17)	0.0256 (8)	
H21	0.9022	0.4594	0.8451	0.031*	

### Atomic displacement parameters (Å^2^)

**Table:**

	*U*^11^	*U*^22^	*U*^33^	*U*^12^	*U*^13^	*U*^23^
O1	0.0233 (12)	0.0323 (14)	0.0259 (12)	0.0001 (10)	0.0093 (9)	−0.0012 (10)
O2	0.0315 (13)	0.0513 (17)	0.0230 (12)	−0.0006 (11)	0.0132 (10)	0.0038 (11)
N1	0.0252 (14)	0.0305 (17)	0.0200 (13)	0.0005 (12)	0.0063 (11)	0.0030 (11)
N2	0.0193 (13)	0.0305 (17)	0.0179 (13)	0.0017 (11)	0.0093 (10)	0.0011 (11)
N3	0.0213 (14)	0.0332 (18)	0.0226 (14)	−0.0016 (12)	0.0080 (11)	0.0057 (12)
C1	0.0251 (17)	0.028 (2)	0.0209 (16)	−0.0040 (14)	0.0075 (13)	−0.0026 (14)
C2	0.0274 (18)	0.034 (2)	0.0242 (18)	−0.0042 (15)	0.0092 (14)	−0.0018 (15)
C3	0.0250 (17)	0.036 (2)	0.0281 (17)	0.0057 (14)	0.0145 (14)	−0.0007 (15)
C4	0.0240 (17)	0.035 (2)	0.0244 (17)	0.0043 (14)	0.0109 (13)	−0.0012 (14)
C5	0.0239 (17)	0.041 (2)	0.0297 (18)	0.0028 (15)	0.0147 (14)	0.0035 (15)
C6	0.0176 (16)	0.044 (2)	0.0348 (19)	−0.0002 (15)	0.0084 (14)	0.0032 (16)
C7	0.0222 (17)	0.038 (2)	0.0293 (18)	0.0033 (15)	0.0059 (13)	0.0009 (15)
C8	0.0256 (17)	0.031 (2)	0.0274 (17)	0.0024 (14)	0.0093 (14)	0.0030 (14)
C9	0.0223 (16)	0.023 (2)	0.0285 (17)	0.0013 (13)	0.0112 (13)	0.0003 (14)
C10	0.0216 (17)	0.026 (2)	0.0253 (17)	0.0004 (13)	0.0084 (13)	−0.0035 (14)
C11	0.0197 (16)	0.027 (2)	0.0193 (15)	−0.0018 (13)	0.0082 (12)	−0.0033 (13)
C12	0.0282 (18)	0.038 (2)	0.0221 (16)	0.0009 (15)	0.0079 (13)	0.0041 (15)
C13	0.0294 (18)	0.040 (2)	0.0183 (16)	−0.0014 (15)	0.0059 (13)	0.0011 (14)
C14	0.0256 (17)	0.036 (2)	0.0279 (17)	0.0005 (15)	0.0063 (14)	0.0023 (15)
C15	0.040 (2)	0.038 (2)	0.036 (2)	−0.0037 (17)	0.0099 (16)	−0.0002 (17)
C16	0.0218 (16)	0.027 (2)	0.0207 (16)	0.0008 (13)	0.0058 (12)	0.0031 (13)
C17	0.0219 (16)	0.034 (2)	0.0220 (16)	0.0008 (14)	0.0060 (13)	0.0016 (14)
C18	0.0276 (17)	0.037 (2)	0.0199 (16)	0.0041 (15)	0.0078 (13)	0.0012 (15)
C19	0.0289 (18)	0.031 (2)	0.0299 (18)	0.0049 (15)	0.0152 (14)	0.0062 (15)
C20	0.0238 (17)	0.030 (2)	0.0285 (17)	−0.0012 (14)	0.0045 (13)	0.0048 (14)
C21	0.0266 (17)	0.029 (2)	0.0227 (16)	0.0023 (14)	0.0091 (13)	0.0024 (14)

### Geometric parameters (Å, °)

**Table:**

O1—C1	1.241 (4)	C10—C11	1.355 (4)
O2—C2	1.218 (4)	C10—C16	1.491 (4)
N1—C1	1.379 (4)	C12—H12A	0.990
N1—C2	1.396 (4)	C12—H12B	0.990
N1—C12	1.459 (4)	C12—C13	1.528 (5)
N2—C2	1.363 (4)	C13—H13A	0.990
N2—C3	1.452 (4)	C13—H13B	0.990
N2—C11	1.417 (4)	C13—C14	1.510 (5)
N3—H3N	0.86 (4)	C14—H14A	0.990
N3—C9	1.413 (4)	C14—H14B	0.990
N3—C10	1.367 (4)	C14—C15	1.519 (5)
C1—C11	1.453 (5)	C15—H15A	0.980
C3—H3A	0.990	C15—H15B	0.980
C3—H3B	0.990	C15—H15C	0.980
C3—C4	1.492 (5)	C16—C17	1.390 (4)
C4—C5	1.396 (5)	C16—C21	1.387 (5)
C4—C9	1.403 (4)	C17—H17	0.950
C5—H5	0.950	C17—C18	1.386 (4)
C5—C6	1.379 (5)	C18—H18	0.950
C6—H6	0.950	C18—C19	1.380 (5)
C6—C7	1.382 (5)	C19—H19	0.950
C7—H7	0.950	C19—C20	1.381 (5)
C7—C8	1.387 (4)	C20—H20	0.950
C8—H8	0.950	C20—C21	1.392 (4)
C8—C9	1.383 (5)	C21—H21	0.950
			
C1—N1—C2	111.6 (3)	C1—C11—C10	128.3 (3)
C1—N1—C12	124.5 (3)	N1—C12—H12A	108.9
C2—N1—C12	123.8 (2)	N1—C12—H12B	108.9
C2—N2—C3	122.9 (2)	N1—C12—C13	113.2 (3)
C2—N2—C11	111.2 (2)	H12A—C12—H12B	107.7
C3—N2—C11	124.5 (3)	H12A—C12—C13	108.9
H3N—N3—C9	115 (2)	H12B—C12—C13	108.9
H3N—N3—C10	115 (2)	C12—C13—H13A	109.2
C9—N3—C10	129.6 (3)	C12—C13—H13B	109.2
O1—C1—N1	122.6 (3)	C12—C13—C14	112.2 (3)
O1—C1—C11	131.4 (3)	H13A—C13—H13B	107.9
N1—C1—C11	105.9 (3)	H13A—C13—C14	109.2
O2—C2—N1	125.6 (3)	H13B—C13—C14	109.2
O2—C2—N2	128.5 (3)	C13—C14—H14A	109.2
N1—C2—N2	105.9 (2)	C13—C14—H14B	109.2
N2—C3—H3A	108.9	C13—C14—C15	111.9 (3)
N2—C3—H3B	108.9	H14A—C14—H14B	107.9
N2—C3—C4	113.4 (3)	H14A—C14—C15	109.2
H3A—C3—H3B	107.7	H14B—C14—C15	109.2
H3A—C3—C4	108.9	C14—C15—H15A	109.5
H3B—C3—C4	108.9	C14—C15—H15B	109.5
C3—C4—C5	120.5 (3)	C14—C15—H15C	109.5
C3—C4—C9	122.2 (3)	H15A—C15—H15B	109.5
C5—C4—C9	117.3 (3)	H15A—C15—H15C	109.5
C4—C5—H5	118.8	H15B—C15—H15C	109.5
C4—C5—C6	122.5 (3)	C10—C16—C17	119.8 (3)
H5—C5—C6	118.8	C10—C16—C21	121.0 (3)
C5—C6—H6	120.4	C17—C16—C21	119.1 (3)
C5—C6—C7	119.2 (3)	C16—C17—H17	119.9
H6—C6—C7	120.4	C16—C17—C18	120.1 (3)
C6—C7—H7	120.2	H17—C17—C18	119.9
C6—C7—C8	119.7 (3)	C17—C18—H18	119.7
H7—C7—C8	120.2	C17—C18—C19	120.6 (3)
C7—C8—H8	119.6	H18—C18—C19	119.7
C7—C8—C9	120.9 (3)	C18—C19—H19	120.2
H8—C8—C9	119.6	C18—C19—C20	119.6 (3)
N3—C9—C4	122.1 (3)	H19—C19—C20	120.2
N3—C9—C8	117.7 (3)	C19—C20—H20	120.0
C4—C9—C8	120.2 (3)	C19—C20—C21	120.1 (3)
N3—C10—C11	126.4 (3)	H20—C20—C21	120.0
N3—C10—C16	113.5 (3)	C16—C21—C20	120.5 (3)
C11—C10—C16	120.1 (3)	C16—C21—H21	119.8
N2—C11—C1	105.0 (2)	C20—C21—H21	119.8
N2—C11—C10	126.6 (3)		
			
C2—N1—C1—O1	−176.4 (3)	C9—N3—C10—C16	158.5 (3)
C2—N1—C1—C11	1.5 (4)	N3—C10—C11—N2	−10.9 (6)
C12—N1—C1—O1	3.5 (5)	N3—C10—C11—C1	165.3 (3)
C12—N1—C1—C11	−178.5 (3)	C16—C10—C11—N2	168.2 (3)
C3—N2—C2—O2	6.5 (5)	C16—C10—C11—C1	−15.6 (5)
C3—N2—C2—N1	−172.0 (3)	C2—N2—C11—C1	5.7 (4)
C11—N2—C2—O2	173.7 (3)	C2—N2—C11—C10	−177.3 (3)
C11—N2—C2—N1	−4.8 (4)	C3—N2—C11—C1	172.7 (3)
C1—N1—C2—O2	−176.7 (3)	C3—N2—C11—C10	−10.4 (5)
C1—N1—C2—N2	2.0 (4)	O1—C1—C11—N2	173.5 (3)
C12—N1—C2—O2	3.4 (5)	O1—C1—C11—C10	−3.4 (6)
C12—N1—C2—N2	−178.0 (3)	N1—C1—C11—N2	−4.2 (3)
C2—N2—C3—C4	−136.8 (3)	N1—C1—C11—C10	178.9 (3)
C11—N2—C3—C4	57.7 (4)	C1—N1—C12—C13	−80.8 (4)
N2—C3—C4—C5	121.8 (3)	C2—N1—C12—C13	99.1 (3)
N2—C3—C4—C9	−61.3 (4)	N1—C12—C13—C14	−180.0 (2)
C3—C4—C5—C6	174.0 (3)	C12—C13—C14—C15	172.6 (3)
C9—C4—C5—C6	−3.1 (5)	N3—C10—C16—C17	−52.2 (4)
C4—C5—C6—C7	−1.3 (5)	N3—C10—C16—C21	127.0 (3)
C5—C6—C7—C8	3.1 (5)	C11—C10—C16—C17	128.6 (3)
C6—C7—C8—C9	−0.3 (5)	C11—C10—C16—C21	−52.2 (5)
C7—C8—C9—N3	174.6 (3)	C10—C16—C17—C18	179.6 (3)
C7—C8—C9—C4	−4.3 (5)	C21—C16—C17—C18	0.3 (5)
C3—C4—C9—N3	9.9 (5)	C16—C17—C18—C19	0.5 (5)
C3—C4—C9—C8	−171.2 (3)	C17—C18—C19—C20	−0.6 (5)
C5—C4—C9—N3	−173.1 (3)	C18—C19—C20—C21	−0.2 (5)
C5—C4—C9—C8	5.8 (5)	C10—C16—C21—C20	179.7 (3)
C10—N3—C9—C4	37.7 (5)	C17—C16—C21—C20	−1.1 (5)
C10—N3—C9—C8	−141.3 (3)	C19—C20—C21—C16	1.1 (5)
C9—N3—C10—C11	−22.3 (6)		

### Hydrogen-bond geometry (Å, °)

**Table:**

*D*—H···*A*	*D*—H	H···*A*	*D*···*A*	*D*—H···*A*
N3—H3N···O1^i^	0.86 (4)	2.10 (4)	2.944 (3)	165 (3)
C8—H8···O1^i^	0.95	2.57	3.326 (4)	136

Symmetry codes: (i) −*x*+3/2, *y*+1/2, −*z*+3/2.
